# Prevalence and Correlates of Peripheral Arterial Disease in Nigerians with Type 2 Diabetes

**DOI:** 10.1155/2016/3529419

**Published:** 2016-10-05

**Authors:** D. O. Soyoye, R. T. Ikem, B. A. Kolawole, K. S. Oluwadiya, R. A. Bolarinwa, O. J. Adebayo

**Affiliations:** ^1^Department of Medicine, Obafemi Awolowo University, Ile-Ife, Nigeria; ^2^Department of Surgery, Ekiti State University, Ado-Ekiti, Nigeria; ^3^Department of Haematology and Immunology, Obafemi Awolowo University, Ile-Ife, Nigeria; ^4^Department of Medicine, Federal Medical Centre, Lokoja, Nigeria

## Abstract

*Background*. Peripheral arterial disease (PAD) is a major risk factor for nonhealing foot ulcers in people with diabetes. A number of traditional risk factors have been reported to be associated with PAD; however, there may be a need to consider nontraditional risk factors especially in some vulnerable populations. This study determined the prevalence and risk factors associated with PAD in diabetics.* Methods*. One hundred and fifty type 2 diabetics and an equal number of age- and sex-matched apparently healthy controls were studied. Assessment of PAD was made using history, palpation of lower limb vessels, and measurement of ankle-brachial index (ABI). Statistically significant differences between categorical and continuous variables were determined using Chi square (*χ* ^2^) and Student* t*-tests, respectively. Regression analysis was done to determine the associated risk factors for PAD.* Results*. Prevalence of PAD using ABI was 22.0% and 8.0% among diabetic and nondiabetic populations, respectively. Peripheral arterial disease was associated with age, male gender, waist circumference, and high-sensitivity C-reactive protein.* Conclusion*. This study highlights the high prevalence of PAD in people with type 2 diabetes mellitus and in apparently healthy controls; age, male gender, abdominal obesity, and high hs-CRP values were the associated risk factors.

## 1. Introduction 

Peripheral arterial disease (PAD) reflects systemic atherosclerosis and is associated with long-term disability and increased cardiovascular complications [1–3]. Lower extremity PAD is the third leading cause of atherosclerotic cardiovascular morbidity after coronary heart disease and stroke, and it is estimated to affect 200 million people globally [4, 5]. Risk factors for PAD, similar to those of other atherosclerotic vascular diseases, include smoking, obesity, diabetes, hypertension, and dyslipidaemia, with smoking and diabetes having the strongest association with PAD [4–8].

Inflammation is known to play a major role in the initiation and progression of atherosclerosis and its complications, and this discovery explains the adoption of inflammatory biomarkers for cardiovascular risk prediction and monitoring [9, 10]. Since the major risk factors such as diabetes and smoking do not explain the increased occurrence of critical limb ischaemia, there is a need to further investigate the contribution of nontraditional factors such as inflammation to peripheral arterial disease. This study assessed the contribution of inflammatory markers, high-sensitivity C-reactive protein (hs-CRP) and white blood cells, to the occurrence of PAD. These have not been previously studied in relation to PAD among indigenous Nigerians. It is hoped that this study will provoke further study of this previously underreported phenomenon in our environment, thus shedding more light on the exact role of these markers in PAD.

## 2. Patients and Methods

One hundred and fifty patients with type 2 diabetes attending the outpatient clinic were selected using systematic sampling in which one in four participants were selected as they registered on clinic days. A total of 150 age- and gender-matched apparently healthy, nondiabetic individuals, who were not being managed for any chronic disease, served as controls. People with ankle-brachial index (ABI) > 1.30, those with febrile illness within one month of study, and those with casts, ulcers, and other conditions that may cause an increase in levels of hs-CRP or interfere with examination of posterior tibial and dorsalis pedis arteries were excluded from the study. Type 2 diabetes mellitus was diagnosed among people who satisfy the World Health Organization (WHO) criteria for the diagnosis of DM and were treated and/or controlled initially using oral hypoglycaemic agents and were being treated with oral hypoglycaemic agents and/or insulin [11, 12].

Peripheral arterial disease (PAD) was defined by the presence of intermittent claudication [13], by detection of two or more reduced or absent pulses of the dorsalis pedis and posterior tibial arteries, with at least one of the legs having both the dorsalis pedis and posterior tibial arteries affected [14–16], and also by an ankle-brachial index (ABI) of ≤0.9 in either of the legs [17]. The lower ABI was used in the analysis. PAD was categorized using ABI into normal (ABI = 0.91–1.30), mild (ABI = 0.70–0.90), moderate (ABI = 0.40–0.69), and severe (ABI < 0.40) [13]. ABI was measured using LifeDop handheld Doppler with 8 Hz probe (Model 150R, Madras Engineering, Chennai).

Baseline information regarding the medical and social history of the respondents was obtained. Blood sample was collected for relevant laboratory investigations: fasting lipid profile, fasting plasma glucose, high-sensitivity C-reactive protein, glycated haemoglobin, and white blood cells count (total and differential). High-sensitivity CRP was measured using enzyme linked immunosorbent assay (Diagnostic Automation, Inc., Calabasas, CA, USA). Total and differential white cell count was done using an automated haematology analyzer (Sysmex America Inc., Mundelein, IL, USA). Written informed consent was sought from and granted by the participants. The study was approved by the Ethics and Research Committee of the hospital. Data was analyzed using SPSS 20. Prevalence of PAD was expressed in percentage. Possible association between categorical variables was determined using Chi square test (*χ* ^2^), and Student's *t-*test was used to detect significant differences between continuous variables. Binary logistic regression analysis was done to determine the associated risks for PAD. The outcome variable was presence or absence of PAD as determined by ABI (lower value of the two legs), while the independent variables were known risk factors for PAD (based on literature review), as well as variables found to be significantly different on bivariate analysis between the diabetic and control groups.* p* value of less than 0.05 was taken as statistically significant.

## 3. Results

A total of 300 subjects were recruited comprising 150 diabetics (66 males and 84 females) and 150 apparently healthy controls (68 males and 82 females) (*p* = 0.908). During screening, 32 patients with diabetes and 12 controls with ABI value >1.3 were excluded from the study. Mean ages of diabetics and controls were 56.12 ± 7.65 years and 55.76 ± 7.49 years, respectively (*p* = 0.681). Thirty-one (10.3%) participants were either current or past smokers; two (0.7%) participants (both controls) were current smokers.  Table 1 compares some demographic, physical, and biochemical characteristics of the diabetic and the control groups.

### 3.1. Prevalence of Peripheral Arterial Disease

Intermittent claudication, a historical indicator of PAD, was present in 27 (18.0%) diabetics and 13 (8.7%) controls (*p* = 0.026). 18 (6.0%) male subjects and 22 (7.3%) female subjects, respectively, had intermittent claudication (*p* = 0.964).

The prevalence of PAD based on palpation method was 19.3% among diabetics and 8.0% among controls (*p* = 0.007). 28 (18.7%) diabetics and 12 (8.0%) controls, respectively, had at least one reduced dorsalis pedis artery pulsation (*p* = 0.011). A similar finding was elicited on examination of the posterior tibial artery: 29 (19.3%) diabetics and 13 (8.7%) controls had reduced pulsation (*p* = 0.018). None of the subjects had absent pulsation.

The prevalence of PAD defined by ABI of <0.9 was 22% in the diabetic group and 8.0% in the control group (*p* = 0.001). Mean ABI for diabetic and control groups were 0.99 ± 0.14 and 1.00 ± 0.08, respectively (*p* = 0.312), with mean ABI in males being 1.00 ± 0.10 (diabetics = 0.99 ± 0.13; controls = 1.00 ± 0.07) and in females 0.99 ± 0.12 (diabetics = 0.98 ± 0.16; controls = 1.00 ± 0.08) (*p* = 0.813). Severity of PAD was graded using ABI, which revealed that 26 (17.3%) diabetics and 12 (8.0%) controls had mild impairment while 7 (4.7%) diabetics had moderate impairment (*p* = 0.001). None of the participants in the control group had moderate impairment and none of the participants in the two groups had severe PAD. The prevalence of PAD increased with age from 0.3% in subjects aged 31 to 40 years to 2.7% in subjects aged 41 to 50 years, 3.3% in subjects aged 51 to 60 years, and 8.7% in subjects aged more than 60 years.

Using ABI as the reference diagnostic method for PAD, the sensitivity and specificity of the historical method were 45% and 94%, respectively. Similarly, the sensitivity and specificity of the palpation method were 80% and 98%, respectively. Twenty-two (14.7%) diabetics and 7 (4.7%) controls satisfied the criteria for diagnosis of PAD using all the three diagnostic methods (*p* = 0.005).

### 3.2. Factors Predicting Occurrence of PAD

There was significant association between age, body mass index (BMI), waist circumference, systolic blood pressure, fasting plasma glucose, serum total cholesterol, serum low-density lipoprotein, serum triglycerides, total white blood cell count, lymphocyte count, serum C-reactive protein, and prevalent PAD, but not with high-density lipoprotein or neutrophil count (Table 2).

Mean ± SD glycated haemoglobin in diabetics who had PAD (8.79 ± 2.37%) was significantly higher than in those without PAD (7.66 ± 1.96%) (*p* = 0.006). Correlation analysis showed negative correlation between glycated haemoglobin and ankle-brachial index (*r* = −0.191, *p* = 0.019). Binary logistic regression showed waist circumference (OR = 13.648; 95% CI = 2.046–91.025; *p* = 0.007), hs-CRP (OR = 1.617; 95% CI = 1.046–2.499; *p* = 0.031), age (OR = 1.110; 95% CI = 1.010–1.221; *p* = 0.031), and male gender (OR = 4.323; 95% CI = 1.153–16.210; *p* = 0.030) as risk factors for PAD in our study population.

## 4. Discussion

Peripheral arterial disease is a reflection of systemic atherosclerosis and diabetes is a major risk factor for atherosclerosis [7, 18, 19]. Prevalence of PAD may vary with age and method of diagnosis and possibly with the gender of the population studied [13, 14, 20]. In this study, we determined the prevalence of peripheral arterial disease using history, palpation method, and ABI measurement.

The prevalence of PAD elicited by history of intermittent claudication was significantly higher in people with diabetes compared with controls. This is similar to what was reported by Bembi et al. [11], who reported prevalence of 16% and 8% in diabetics and controls, respectively. Intermittent claudication may not be a true indicator of PAD due to the presence of physical impairment and cardiovascular diseases in people with PAD, resulting in their inability to walk far or fast enough to experience muscle ischaemia which brings about intermittent claudication [21, 22]. This may particularly apply among the elderly, where the presence of other conditions such as osteoarthritis may also affect walking [23]. In addition, conditions such as degenerative joint disease, spinal stenosis, and herniated disc may also cause exertional leg pain; hence, intermittent claudication may be nonspecific [20].

The prevalence of PAD using palpation was 19.3% among people with diabetes, which was significantly higher than the 8.0% obtained among controls. More subjects had reduced pulsation of posterior tibial artery compared with dorsalis pedis artery, and none of the subjects had absent pulsation. It is known that dorsalis pedis artery may be absent in a proportion of normal individuals [24, 25]; this was not the case in our study.

Prevalence of PAD (defined by an ABI of <0.9%) was 22.0% and 8% among the diabetics and controls, respectively; none of our participants had severe PAD. The prevalence rates were similar to results obtained by Bembi et al. [11], who obtained prevalence rates of 24% and 8%, respectively, in diabetics and controls. The prevalence rate of PAD in our study is also similar to that reported by Beach et al. [26], who examined people aged 50–70 years using ABI. They found prevalence of 22% among diabetics compared with 3% among controls [26]. We excluded people with leg ulcer; this could inadvertently exclude people with severe arterial disease, and this may explain the absence of people with absent peripheral pulses on palpation and severe PAD on ABI estimation among our study participants.

The prevalence of PAD varied, depending on the method utilized in this study. This is similar to the findings of Ogbera et al. [27] who obtained values of 10.7% for each of intermittent claudication and palpation and 12% for ABI in diabetics with foot at risk. The prevalence in this study was lower than that obtained in our earlier study [16] where we obtained prevalence of PAD of 25.7% and 55.4% on pulse palpation and ABI, respectively [16]. The disparity between this and our earlier study may be explained by the fact that most (62.2%) of the patients reviewed in our earlier study already had foot ulcer, which is one of the major complications of PAD, hence the higher prevalence rates of PAD. Participants with ulcer were excluded from our present study. Palpation may be affected by such factors as ambient temperature, anatomical variations, and interobserver differences. The presence of claudication and an abnormal pulse may therefore underestimate the prevalence of PAD [14, 28].

Inaccurate measurements from ankle-brachial index studies may occur due to the presence of calcified or incompressible vessels (which would produce falsely elevated readings), especially in diabetic and elderly patients, and subclavian-artery stenosis [15]. Despite these limitations, ABI measurement has a sensitivity above 90% in contrast to intermittent claudication with a sensitivity of about 50% [15, 24] and a specificity of 95% for the diagnosis of peripheral arterial disease [15]. It has also been reported to have marginal observer variability [24]. In our study, the sensitivity and specificity of intermittent claudication were 45% and 94%, respectively, and the sensitivity and specificity of the palpation method were 80% and 98%, respectively, with reference to the ankle-brachial index. History of intermittent claudication and detection of reduced peripheral pulses are diagnostic indicators of peripheral arterial disease and are relevant in clinical practice, but the use of ankle-brachial index should be more sensitive in detecting PAD.

Early risk factor identification and treatment may be a cost-effective means of preventing peripheral arterial disease and its complications. Previous studies [17, 24, 29] have suggested the association of PAD with both modifiable and nonmodifiable risk factors. The most common risk factors associated with PAD were advanced age, diabetes, and smoking [24, 26, 30].

As previously reported [31], our study also showed significant association between waist circumference (WC) and PAD, with an almost fourteenfold risk of peripheral arterial disease with increasing waist circumference (OR = 13.648; 95% CI = 2.046–91.025). Also, WC values were significantly higher in diabetics compared with controls. Waist circumference is a measure of central obesity (adiposity), and adipocytes function as an endocrine organ releasing some cytokines and adiponectins such as the tumour necrosis factor (TNF) *α*, leptin, interleukin-6 (IL-6), prothrombotic agents such as plasminogen activator inhibitor 1 (PAI-1), and angiotensinogen [32, 33]. These factors have their roles in inflammation, coagulation, and atherogenesis [32, 33]. In addition, abdominal obesity is also associated with an atherogenic lipid profile [32].

The level of hs-CRP was higher among diabetics compared with controls. There is about twofold risk of PAD with increasing levels of hs-CRP (OR = 1.617; 95% CI = 1.046–2.499). This finding is consistent with previous reports [17, 34], demonstrating significant association between CRP and PAD. CRP is a marker of inflammation, a characteristic of all phases of atherothrombosis [9, 10, 35]. CRP is also postulated to directly influence vascular vulnerability and progression of atherosclerosis through several mechanisms, including enhanced expression of local adhesion molecules, increased expression of endothelial plasminogen activator inhibitor 1 (PAI-1), reduced endothelial nitric oxide bioactivity, altered low-density lipoprotein (LDL) uptake by macrophages, and colocalization with complement within atherosclerotic lesions [9, 10, 35].

Advancing age was another associated risk for PAD in this study, which was also reported as a risk in previous studies [11, 17, 36]. Association of PAD with advancing age may be explained by the fact that the incidence of other risk factors associated with atherosclerosis, such as diabetes and hypertension, also increases with age [12, 37]. These risks are further accentuated by physical inactivity which is worsened by degenerative joint disease in the elderly. The favourable lipoprotein pattern and reduced incidence of atherosclerosis in premenopausal women is also lost after menopause [38].

Male sex increased the risk of PAD in this study more than four times (OR = 4.323; 95% CI = 1.153–16.210), a similar finding in other studies [22, 36]. Increased prevalence of PAD in males may be due to the relatively lower levels of high-density lipoprotein (HDL) in men compared with women [38]. The protective role of HDL may be due to its role in augmenting peripheral catabolism of cholesterol via the reverse cholesterol transfer and its carriage of antioxidant enzymes which reduce the level of oxidized phospholipids in atheromatous lesions [35].

There was a significant negative correlation between glycated haemoglobin (a measure of long-term glycaemic control) and ABI (*r* = −0.191, *p* = 0.019). Glycated haemoglobin level was also significantly higher among diabetics who had PAD compared with diabetics without PAD. This association between PAD and hyperglycaemia has been found in several studies [36, 39, 40]. Hyperglycaemia may potentiate atherogenesis by inhibiting arterial endothelial nitric oxide (NO) production, enhancing platelet-derived growth factor- (PGDF-) induced vascular smooth muscle cell proliferation, stimulating PAI-1 production, and accumulating advanced glycation end products [6, 35]. In addition, hyperglycaemia, especially in the setting of insulin resistance, may be associated with atherogenic diabetic dyslipidaemia [38].

Smoking has been reported to be an important risk for PAD [17, 24, 26, 29]; however, smoking was not a significant factor in this study. The reason for this may be that few subjects (10.3%) in both diabetic (less than 15%) and control (6%) groups ever smoked. This smoking rate compares with a rate of 7.0% of adult Nigerians who ever smoked reported in Global Adult Tobacco Survey (GATS) Nigeria [41]. Smoking cessation counseling, which is part of diabetes care, may explain the absence of current smokers among the diabetics. A dose-response relationship exists between pack-year history and PAD risk [26]. None of the subjects actively smoked during the time of the study, and most smoked less than five (5) pack-years. Hence, the smaller number of smokers and the minimal smoking dose in those that smoked may explain the lack of association between smoking and PAD that we observed.

## 5. Conclusion

The prevalence of peripheral arterial disease is high among diabetic patients and may be a major contributor to lower limb morbidities. Early detection and reliable diagnosis can be made using simple methods such as palpation and measurement of ankle-brachial index. We have shown that peripheral arterial disease is associated with increasing age, male gender, abdominal obesity, and high hs-CRP levels, suggesting that strategies to reduce the occurrence of PAD in people with type 2 diabetes mellitus should not only focus on controlling traditional risk factors, but also consider the effects of inflammation. Future research into strategies aimed at controlling inflammation among people with type 2 diabetes should be considered.

## Figures and Tables

**Table 1:** Comparison of traditional and nontraditional factors among diabetics and controls.

Parameter	Diabetics	Controls	*p* value
	*N* = 150	*N* = 150	
Smokers (past & present)	22 (14.7%)	9 (6.0%)	0.368
<5 pack-years	16 (10.7%)	9 (6.0%)	0.986
>5 pack-years	6 (4.0%)	0 (0%)	
Alcohol intake (individuals)	38 (25.3%)	19 (12.6%)	0.039
BMI (kg/m^2^)	27.8 ± 4.8	25.8 ± 4.7	<0.001
WC (cm)	95.9 ± 11.7	88.8 ± 9.4	<0.001
SBP	132.8 ± 17.6	117.7 ± 11.2	<0.001
FPG (mmol/L)	7.7 ± 3.5	4.4 ± 0.7	<0.001
HbA1c (%)	7.9 ± 2.1	ND	
TC (mmol/L)	4.3 ± 0.9	3.8 ± 0.7	<0.001
HDL (mmol/L)	1.0 ± 0.2	1.1 ± 0.3	0.001
LDL (mmol/L)	2.8 ± 0.8	2.6 ± 0.7	0.009
hs-CRP (mg/L)	1.49 ± 1.64	0.74 ± 0.82	<0.001
Total white cell count (×10^3^)	5.90 ± 1.21	4.97 ± 0.99	<0.001
Lymphocyte count (×10^3^)	2.68 ± 0.83	2.37 ± 0.65	<0.001
Neutrophil count (×10^3^)	3.16 ± 0.87	2.57 ± 0.80	<0.001

BMI: body mass index; WC: waist circumference; WHR: waist-to-hip ratio; SBP: systolic blood pressure; FPG: fasting plasma glucose; HbA1c: glycated haemoglobin; ND: not done; TC: total cholesterol; HDL: high-density lipoprotein; LDL: low-density lipoprotein; hs-CRP: high-sensitivity C-reactive protein.

**Table 2:** Association of traditional and nontraditional risk factors and peripheral arterial disease.

Variable	Subjects with PAD	Subjects without PAD	*p* value
	(*n* = 45)	(*n* = 255)	
	Mean values ± SD		
Age (years)	58.24 ± 7.73	55.53 ± 7.47	0.033
BMI (kg/m^2^)	29.07 ± 5.92	26.39 ± 4.48	0.005
WC (cm)	100 ± 13.09	90.95 ± 10.28	<0.001
SBP (mmHg)	133.16 ± 22.03	123.81 ± 14.97	0.009
FPG (mmol/L)	7.52 ± 4.10	5.74 ± 2.66	0.007
TC (mmol/L)	4.62 ± 1.24	3.98 ± 0.79	0.001
HDL (mmol/L)	1.01 ± 0.21	1.04 ± 0.23	0.429
LDL (mmol/L)	3.09 ± 0.96	2.63 ± 0.71	0.003
TG (mmol/L)	1.01 ± 0.35	0.90 ± 0.33	0.046
Total white cell (×10^3^)	6.16 ± 1.53	5.31 ± 1.08	0.001
Neutrophil count	3.00 ± 1.12	2.84 ± 0.84	0.381
Lymphocyte count	3.11 ± 1.15	2.42 ± 0.62	<0.001
hs-CRP (mg/L)	1.83 ± 1.12	0.88 ± 1.08	<0.001

PAD: peripheral arterial disease; BMI: body mass index; WC: waist circumference; SBP: systolic blood pressure; DBP: diastolic blood pressure; FPG: fasting plasma glucose; HDL: high-density lipoprotein; LDL: low-density lipoprotein; hs-CRP: high-sensitivity C-reactive protein.
